# Combined detection of SIL-2R, VEGF, and ES for predicting recurrence in papillary thyroid carcinoma: Correlation with estrogen levels

**DOI:** 10.5937/jomb0-59085

**Published:** 2025-11-05

**Authors:** Ci Xin, Shaoyu Han, Jianli Cui, Yan Guo

**Affiliations:** 1 Changchun University of Chinese Medicine, College of Integrated Chinese and Western Medicine, Changchun, Jilin, 130117, China; 2 Sichuan Taikang Hospital, Department of Laboratory Medicine, Chengdu, Sichuang, 611400, China

**Keywords:** Papillary thyroid cancer, prognostic recurrence, sIL-2R, VEGF, ES, diagnosis, Papilarni karcinom štitne žlezde, prognostički recidiv, sIL-2R, VEGF, ES, dijagnoza

## Abstract

**Background:**

To explore the correlation of serum soluble interleukin-2 receptor (sIL-2R), vascular endothelial growth factor (VEGF), and endostatin (ES) with estrogen levels in papillary thyroid carcinoma (PTC) patients, and to assess the predictive efficacy of these biomarkers for PTC diagnosis and postoperative recurrence.

**Methods:**

From March 2023 to March 2024, 132 newly diagnosed PTC patients and 128 healthy controls were enrolled. Serum sIL-2R, VEGF, and ES levels were quantified using enzyme-linked immunosorbent assay (ELISA), while estrogen levels (estrone [E1], estradiol [E2], estriol [E3]) were measured via chemiluminescent immunoassay. Patients were followed postoperatively for one year to monitor recurrence events, including local recurrence, lymph node metastasis, and distant metastasis. The diagnostic performance of the combined model was evaluated using receiver operating characteristic (ROC) curve analysis, and Pearson correlation analysis was conducted to examine the relationship between biomarkers and estrogen levels.

**Results:**

Compared to controls, PTC patients exhibited significantly elevated serum sIL-2R, VEGF, and ES levels (P&lt; 0.05). The combined detection of these biomarkers demonstrated a sensitivity of 80.30% and specificity of 78.91% (AUC= 0.8526) for PTC diagnosis. Additionally, E1 and E2 levels were significantly higher in PTC patients (P&lt; 0.05) and showed positive correlations with sIL-2R, VEGF and ES (P&lt; 0.05), whereas E3 levels changed insignificantly (P&gt; 0.05). Recurrent patients had significantly higher sIL-2R, VEGF and ES levels than non-recurrent patients (P&lt; 0.05). The combined predictive model for recurrence achieved a sensitivity of 96.88% and specificity of 61.00% (AUC= 0.8494).

**Conclusions:**

Elevated serum sIL-2R, VEGF, and ES levels in PTC patients indicate that their combined assessment may serve as a sensitive and specific tool for PTC diagnosis and postoperative recurrence risk stratification (AUC = 0.8494).

## Abstract (sr)

**Uvod:**

Cilj istraživanja je ispitivanje korelacije serumski rastvorljivog receptora za interleukin-2 (sIL-2R), vaskularnog endotelnog faktora rasta (VEGF) i endostatina (ES) sa nivoima estrogena kod pacijenata sa papilarnim karcinomom štitne žlezde (PTC), kao i procena prediktivne efikasnosti ovih biomarkera za dijagnozu PTC i postoperativni recidiv.

**Metode:**

Od marta 2023. do marta 2024. godine, uključeno je 132 novodijagnostikovana pacijenta sa PTC i 128 zdravih kontrola. Nivoi serumskih sIL-2R, VEGF i ES kvantifikovani su pomoću enzimski povezanog imunosorbentnog testa (ELISA), dok su nivoi estrogena (estron [E1], estradiol [E2], estriol [E3]) mereni hemiluminiscentnim imunotestom. Pacijenti su praćeni postoperativno godinu dana radi praćenja recidiva, uključujući lokalni recidiv, metastaze limfnih čvorova i udaljene metastaze. Dijagnostičke performanse kombinovanog modela procenjene su pomoću analize ROC krive, a sprovedena je Pirsonova korelaciona analiza kako bi se ispitao odnos između biomarkera i nivoa estrogena.

**Rezultati:**

U poređenju sa kontrolnom grupom, pacijenti sa PTC su pokazali značajno povišene nivoe sIL-2R, VEGF i ES u serumu (P&lt; 0,05). Kombinovana detekcija ovih biomarkera pokazala je osetljivost od 80,30% i specifičnost od 78,91% (AUC= 0,8526) za dijagnozu PTC. Pored toga, nivoi E1 i E2 bili su značajno viši kod pacijenata sa PTC (P&lt; 0,05) i pokazali su pozitivne korelacije sa sIL-2R, VEGF i ES (P&lt; 0,05), dok su se nivoi E3 beznačajno promenili (P&gt; 0,05). Pacijenti sa recidivom imali su značajno više nivoe sIL-2R, VEGF i ES nego pacijenti bez recidiva (P&lt; 0,05). Kombinovani prediktivni model za recidiv postigao je osetljivost od 96,88% i specifičnost od 61,00% (AUC= 0,8494).

**Zaključak:**

Povišeni nivoi sIL-2R, VEGF i ES u serumu kod pacijenata sa PTC ukazuju na to da njihova kombinovana procena može poslužiti kao osetljiv i specifičan alat za dijagnozu PTC i stratifikaciju rizika od postoperativnog recidiva (AUC = 0,8494).

## Introduction

Papillary thyroid carcinoma (PTC), the most common histological subtype of differentiated thyroid cancer, represents more than 80% of all thyroid malignancies [1]. While PTC is generally associated with a favorable prognosis, demonstrating a 10-year postoperative survival rate exceeding 90%, disease recurrence—particularly local recurrence and lymph node metastasis—remains a significant clinical challenge that adversely impacts long-term patient outcomes [2]. Current postoperative risk stratification for PTC predominantly depends on conventional histopathological characteristics (e.g., tumor size, lymph node metastasis [LNM], and extrathyroidal invasion) and serum biomarkers (such as thyroglobulin levels). However, the diagnostic performance of these parameters is suboptimal, with inherent limitations in both sensitivity and specificity, thereby hampering the precise identification of high-risk individuals [3]. Consequently, there is an urgent clinical imperative to discover and validate novel biomarkers with superior predictive capabilities to refine risk assessment protocols and facilitate personalized therapeutic strategies for PTC patients.

In recent years, the tumor microenvironment and immune-inflammatory responses have garnered considerable attention for their pivotal roles in cancer development and progression [4]. Soluble interleukin-2 receptor (sIL-2R), a well-established marker of T-cell activation, demonstrates elevated levels that correlate with immune evasion mechanisms across various solid tumors [5]. Concurrently, vascular endothelial growth factor (VEGF) and endostatin (ES), a critical cytokine pair governing angiogenesis, have been shown to significantly influence tumor proliferation and invasive potential [6] [7]. Notably, a study by Pangarsa et al. on diffuse large B-cell lymphoma revealed a novel regulatory relationship between sIL-2R and VEGF [8], suggesting potential synergistic interactions among sIL-2R, VEGF, and ES in tumor biology. Despite these advances, the mechanistic interplay between sIL-2R, VEGF, and ES levels in PTC remains poorly understood. Furthermore, there is a notable gap in systematic investigations evaluating the combined predictive efficacy of these biomarkers for postoperative recurrence in PTC patients.

Epidemiological studies have consistently demonstrated a striking gender disparity in the incidence of PTC, with females comprising approximately 75% of cases [9]. This pronounced female predominance strongly implicates estrogen in PTC pathogenesis, potentially through its modulation of inflammatory and angiogenic pathways within the tumor microenvironment. Based on these findings, our study introduces an innovative approach by systematically integrating immune-inflammatory markers (sIL-2R), angiogenesis-related factors (VEGF, ES), and estrogen levels into a unified analytical framework. This comprehensive strategy enables us to elucidate the dynamic interplay among these biomarkers in PTC patients and to develop a novel multi-marker predictive model for postoperative recurrence. Notably, this investigation represents the first systematic exploration of both the individual and synergistic prognostic value of these biomarkers, thereby overcoming the inherent limitations of conventional single-marker analyses. Should the clinical utility of this model be validated, our findings could yield two major advancements: (1) providing novel molecular insights into the mechanisms underlying PTC recurrence, and (2) establishing evidence-based protocols for the early identification, longitudinal monitoring, and targeted management of high-risk postoperative patients. Such advancements would facilitate more precise risk stratification, mitigating the risks of either overtreatment or delayed intervention, and ultimately contribute to the refinement of precision medicine strategies in PTC management.

## Materials and methods

### Study design

We conducted a prospective study involving patients diagnosed with PTC admitted to our hospital between March 2023 and March 2024. Using G*Power 3.1 (effect size=0.3, a = 0.05, power= 0.95), we first estimated a minimum required sample size of 111 subjects. Initially, 184 PTC patients were screened based on the following inclusion criteria: Diagnosis confirmed by pathological examination and meeting standard diagnostic criteria for PTC [10]; treatment-naïve, primary PTC; female, age >18 years; unilateral disease; complete clinical data available. After applying exclusion criteria, 132 patients were included in the final analysis. Exclusions were based on comorbid mental disorders, concurrent Hashimoto's thyroiditis or hyperthyroidism, coagulation dysfunction or uncontrolled systemic diseases, history of other malignancies, prior neck/chest surgery or radiotherapy, organ dysfunction, and loss to follow-up. Additionally, 128 age-matched healthy controls from the same period were enrolled for comparison. Baseline characteristics of both groups are presented in Table 1.

**Table 1:** Comparison of clinical data of control group and PTC patients.

	Age (years)	Family history<br>of PTC	Smoking	Drinking	Diameter of<br>tumor (cm)	Location of<br>the lesion<br>Left /right
Control group<br>(n = 128)	54.82±8.17	9 (7.03%)	30 (23.44%)	13 (10.16%)	-	-
PTC patients<br>(n = 132)	55.14±5.90	12 (9.09%)	35 (26.52%)	15 (11.36%)	1.37±0.35	72 (54.55%)/<br>60 (45.45%)
t	0.367	0.371	0.328	0.099	-	-
P	0.714	0.542	0.567	0.754	-	-

### Ethical considerations

This study was approved by the Institutional Ethics Committee and conducted in compliance with the Declaration of Helsinki. Written informed consent was obtained from all participants.

### Methods

Two groups of study subjects had 4-5 mL of fasting blood collected from their elbow veins in the morning upon admission to the hospital and placed in anticoagulant tubes. After collection, samples were allowed to clot at room temperature for 30-60 minutes before centrifugation (1,505 X g, 4°C, 15 min) to separate serum. The obtained serum was aliquoted and stored at -80°C until analysis. Serum concentrations of sIL-2R, VEGF, and ES were quantified using commercial ELISA kits according to the manufacturers' protocols. Briefly, standard solutions were prepared using serial dilutions covering the following ranges: sIL-2R (50-3200 pg/mL), VEGF (15.6-1000 pg/mL), and ES (0.5-32 ng/mL). Serum samples were appropriately diluted when necessary. For the assay, a volume of 100 μL was added to each well, followed by incubation at 37°C for 90 minutes. After washing, detection antibodies were added, followed by streptavidin-HRP conjugate and TMB substrate. The reaction was then stopped, and absorbance (optical density, OD) was measured at 450 nm. Sample concentrations were determined from the standard curve, with the coefficient of variation (CV) for duplicate wells being <10%. Each assay plate included blank wells, negative controls, and quality controls, with inter-assay variation maintained below 15%. Additionally, estrogen (estrone [E1], estradiol [E2], estriol [E3]) levels were measured using a fully automated chemiluminescence analyzer with manufacturer-matched reagent kits. Samples were processed according to standard protocols, including incubation at 37°C, with quantification based on chemiluminescence signal intensity.

### Prognostic follow-up

All PTC patients underwent prognostic follow-up for at least one year after discharge, conducted through scheduled re-examinations. Monthly for first 6 months, then every two months thereafter. The study documented any cases of PTC recurrence, which included the following: (1) Local recurrence: the reappearance of the tumor in the thyroid bed, residual thyroid tissue, or adjacent cervical structures following complete surgical resection of the primary tumor; (2) LNM: the dissemination of tumor cells to regional lymph nodes, resulting in the formation of new metastatic foci; (3) Distant metastasis: the spread of tumor cells to other parts of the body. In terms of imaging, (1) Local recurrence: Hypoechoic nodule 10 mm in thyroid bed on ultrasound (TI-RADS 4B/5). (2) Lymph node metastasis: Cervical node 10 mm short-axis with loss of fatty hilum + hypervascularity. (3) Distant metastasis: PET-CT SUVmax >2.5 in extra-thyroidal sites.

### Observation indicators

Serum levels of sIL-2R, VEGF, ES, and estrogen were compared between PTC patients and healthy controls. The diagnostic efficacy of combined detection (sIL-2R+VEGF + ES) for PTC was evaluated using receiver operating characteristic (ROC) curve analysis. Subsequently, differences in sIL-2R, VEGF, and ES levels were assessed between recurrent and nonrecurrent PTC patients during follow-up, and the prognostic value of these markers for predicting recurrence was analyzed.

### Statistical analysis

All statistical analyses were performed using SPSS 25.0 (IBM Corp.). Categorical variables, including family medical history and lesion location [presented as n (%)], were compared using the chi-square test. Continuous variables, such as sIL-2R and VEGF expression levels, were first evaluated for normality using the Shapiro-Wilk test. Normally distributed data were expressed as (χ̄±s) and analyzed using independent samples t-tests. Pearson's correlation coefficient was employed to assess relationships between variables. Diagnostic and prognostic performance was determined through ROC curve analysis. For joint testing, logistic regression analysis is used to obtain the joint formula. A P-value<0.05 was considered statistically significant.

## Results

### Association of sIL-2R, VEGF, and ES with PTC

**Figure 1:**
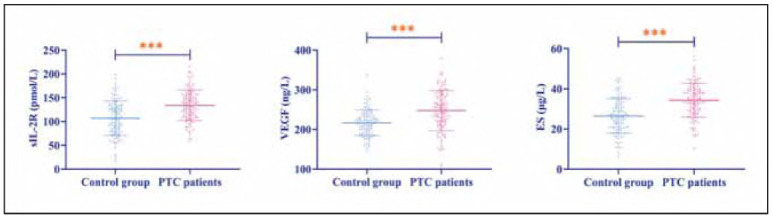
Comparison of sIL-2R, VEGF and ES expression levels in control and PTC patients. ***P<0.001.

Compared to controls, PTC patients exhibited significantly elevated serum sIL-2R, VEGF, and ES expression (P<0.001), suggesting their potential involvement in PTC pathogenesis and progression (Figure 1).

### Diagnostic utility of sIL-2R, VEGF, and ES in PTC

**Figure 2:**
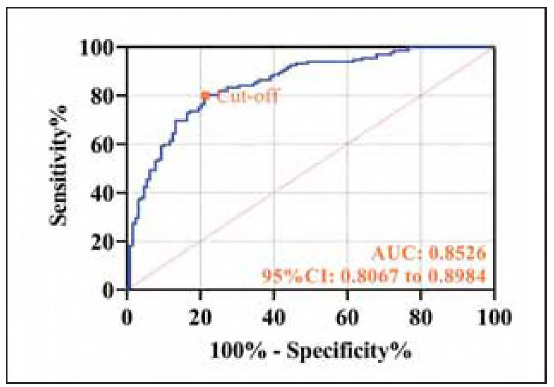
ROC curve of sIL-2R, VEGF, and ES combined test for diagnosis of PTC.

High sIL-2R, VEGF, and ES levels were significantly associated with an increased risk of PTC (P<0.001, Table 2). A predictive model based on regression coefficients was established: Y_prediction_ = - 10.189+0.027xsIL-2R+0.016xVEGF+0.111xES. ROC curve analysis demonstrated that the combined use of these three biomarkers achieved a sensitivity of 80.30% and a specificity of 78.91% (P<0.001, Figure 2).

**Table 2:** Relationship between sIL-2R, VEGF ES and PTC.

	B		Wals	df	Sig.	Exp (B)	95%CI
sIL-2R	0.027	0.005	29.376	1	<0.001	1.027	1.017-1.037
VEGF	0.016	0.004	17.610	1	<0.001	1.016	1.009-1.024
ES	0.111	0.019	33.343	1	<0.001	1.117	1.076-1.160
Constant	-10.189	1.331	58.592	1	<0.001	-	-

### Correlation of sIL-2R, VEGF, and ES, with estrogen levels

Regarding estrogen levels, PTC patients exhibited higher levels of E1 and E2 compared to controls (P<0.050), whereas E3 levels showed no significant difference (P=0.267). Pearson correlation analysis revealed that sIL-2R, VEGF, and ES in PTC patients showed no significant correlation with E3 (P>0.050) but were positively correlated with E1 and E2 (P<0.001, Table 3).

**Table 3:** Comparison of estrogen levels of control group and PTC patients.

	E1 (pg/mL)	E2 (pg/mL)	E3 (pg/mL)
Control group (n = 128)	31.93±12.21	7.51±2.94	1.21±0.30
PTC patients (n=132)	34.98±8.62	9.77±3.18	1.26±0.38
t	2.334	5.950	1.113
P	0.020	<0.001	0.267
Pearson's correlation coefficient (r/P)			
	E1 (pg/mL)	E2 (pg/mL)	E3 (pg/mL)
sIL-2R (pmol/L)	0.479/<0.001	0.503/<0.001	-0.035/0.692
VEGF (ng/L)	0.581/<0.001	0.533/<0.001	-0.113/0.197
ES (μg/L)	0.522/<0.001	0.484/<0.001	-0.066/0.451

### Association of sIL-2R, VEGF, and ES with PTC recurrence

Postoperative sIL-2R/VEGF/ES levels decreased significantly compared to preoperative levels (P<0.010). During follow-up, the 1-year recurrence rate in PTC patients was 24.24% (32/132). Comparative analysis revealed that patients who experienced recurrence had significantly higher levels of sI L-2R, VEGF, and ES than those without recurrence (P<0.010).

### Prognostic value of sIL-2R, VEGF, and ES for PTC recurrence

**Figure 3:**
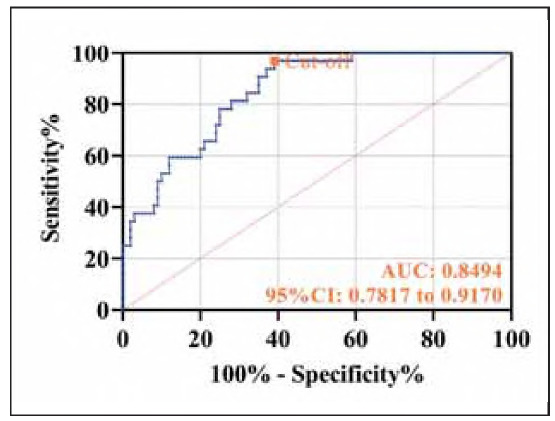
ROC curve of sIL-2R, VEGF, and ES combined test for diagnosis of prognostic recurrence of PTC.

Similarly, regression analysis identified high expression of sIL-2R, VEGF, and ES as independent risk factors for PTC recurrence (P<0.050, Table 4). The joint prediction equation was Y_prediction_ = - 15.089 + 0.026xsIL-2R + 0.025xVEGF + 0.152 xE, which demonstrated a diagnostic sensitivity of 96.88% and specificity of 61.00% for 1-year recurrence (P<0.001, Figure 3).

**Table 4:** Relationship between sIL-2R, VEGF, ES and prognostic recurrence of PTC.

	B	S.E.	Wals	df	Sig.	Exp (B)	95%CI
sIL-2R	0.026	0.010	6.678	1	0.010	1.027	1.006-1.048
VEGF	0.025	0.007	13.303	1	<0.001	1.025	1.011-1.039
ES	0.152	0.040	14.311	1	<0.001	1.165	1.076-1.260
Constant	-15.089	2.852	27.992	1	<0.001	-	-

## Discussion

The present study demonstrates that sIL-2R, VEGF, and ES exhibit strong diagnostic performance in predicting the occurrence and prognostic recurrence of PTC. These findings offer valuable insights for future PTC disease assessment and clinical decision-making.

First, our findings demonstrated significantly elevated levels of E1 and E2 in PTC patients, which exhibited a positive correlation with sIL-2R, VEGF, and ES. In contrast, E3 showed no significant differences. These results lend support to the hypothesis that estrogen-particularly E2-may facilitate PTC progression by modulating inflammatory and angiogenic pathways [11]. Mechanistically, estrogen likely binds to estrogen receptors (ERα/ERβ), triggering activation of the PI3K/AKT or MAPK signaling cascades. Prior studies indicate estrogen-mediated PI3K/AKT activation upregulates VEGF in breast cancer [12], suggesting a plausible mechanism in PTC. Furthermore, the observed association between estrogen and sIL-2R implies a potential role in reshaping the tumor immune microenvironment through T-cell regulation [13]. Collectively, these findings suggest that estrogen may potentially drive activation of the inflammation-angiogenesis axis and underscores the therapeutic potential of targeting estrogen signaling pathways in PTC management. Nevertheless, the precise biological role of E3 in PTC pathogenesis warrants further investigation.

Additionally, we observed significantly elevated serum levels of sIL-2R, VEGF, and ES in PTC patients, indicating their potential involvement in PTC progression through distinct mechanisms. As a marker of T-cell activation, elevated sIL-2R likely reflects persistent immune-inflammatory responses within the tumor microenvironment, which may contribute to immune evasion and tumor cell survival [14]. We hypothesize that the marked increase in sIL-2R among PTC patients could suppress local anti-tumor immune responses, thereby indirectly facilitating disease progression [15]. Furthermore, VEGF, a key pro-angiogenic factor, is closely associated with enhanced PTC invasiveness when overexpressed [16]. While ES, an endogenous angiogenesis inhibitor, may rise as a compensatory regulatory response to tumor angiogenesis [17]. The concurrent elevation of VEGF and ES in PTC patients suggests that, despite the compensatory rise in ES to counteract excessive VEGF activity, the overall process remains dominated by proangiogenic signaling [18]. Correlative data suggest potential crosstalk between sIL-2R and VEGF/ES pathways, though mechanistic validation is needed [19].

Traditional biomarkers such as thyroglobulin (Tg) have notable limitations in the postoperative monitoring of PTC, including suboptimal sensitivity and susceptibility to antibody interference [20]. In this study, we propose for the first time a novel combined detection model incorporating sIL-2R, VEGF, and ES, which demonstrates superior diagnostic efficacy for PTC recurrence (AUC = 0.8526). This finding underscores the potential of integrating multidimensional biomarkers to more comprehensively capture tumor biological characteristics. Notably, all three biomarkers exhibited significantly elevated levels in patients with recurrence compared to those without. The combined model showed robust predictive performance for 1-year recurrence, achieving a sensitivity of 96.88% and a specificity of 61.00%. These results provide a promising strategy for the early identification of high-risk postoperative patients, aligning with the »multi-parameter risk stratification« framework proposed by Kong X et al. [21], which emphasizes optimizing individualized surveillance intervals and therapeutic strategies through multi-biomarker analysis.

Based on the findings of this study, we recommend integrating the combined detection of sIL-2R, VEGF, and ES into the postoperative stratified management protocol for PTC, particularly for dynamic monitoring of high-risk patients. The combined ELISA panel is clinically feasible (turnaround <6h). Automated platforms (e.g., Meso Scale Discovery) could further streamline testing for routine monitoring. Furthermore, for patients exhibiting elevated sIL-2R/VEGF levels, potential adjuvant therapeutic approaches like anti-inflammatory agents (e.g., JAK inhibitors tofacitinib) or anti-angiogenic therapies (e.g., anti-VEGF antibody bevacizumab) warrant further investigation. Nevertheless, this study has several limitations: First, the relatively small sample size and short follow-up duration (1 year) may lead to an underestimation of long-term recurrence risks. Second, the potential influence of menopausal status on estrogen levels was not evaluated through stratified subgroup analysis. Finally, the mechanistic investigation was confined to correlation analyses without in vitro or in vivo experimental validation. Lack of T-cell subset profiling (e.g., Treg/Th17 ratios) limits mechanistic interpretation of sI L-2R data. Future studies should integrate immune contexture analysis to clarify how estrogen-driven inflammation modulates tumor immunity.

## Conclusion

The synergistic upregulation of sI L-2 R, VEGF, and ES represents a distinctive hallmark of both PTC pathogenesis and disease recurrence. Their combined assessment serves as a robust clinical stratification tool for risk evaluation. Our findings suggest that estrogen (notably E2) may promote tumor progression through the inflammation-angiogenesis axis, suggesting potential value of targeting estrogen pathways.

## Dodatak

### Availability of data and materials

The data that support the findings of this study are available from the corresponding author upon reasonable request.

### Acknowledgements

Not applicable.

### Conflict of interest statement

All the authors declare that they have no conflict of interest in this work.
